# Evaluation of Revised US Preventive Services Task Force Lung Cancer Screening Guideline Among Women and Racial/Ethnic Minority Populations

**DOI:** 10.1001/jamanetworkopen.2020.33769

**Published:** 2021-01-12

**Authors:** Thomas J. Reese, Chelsey R. Schlechter, Lindsey N. Potter, Kensaku Kawamoto, Guilherme Del Fiol, Cho Y. Lam, David W. Wetter

**Affiliations:** 1 Department of Biomedical Informatics, University of Utah, Salt Lake City; 2 Department of Population Health Sciences, University of Utah, Salt Lake City; 3 Huntsman Cancer Institute, University of Utah, Salt Lake City

## Abstract

**Importance:**

Lung cancer incidence and mortality disproportionately affect women and racial/ethnic minority populations, yet screening guidelines for the past several years were derived from clinical trials of predominantly White men. To reflect current evidence, the US Preventive Services Task Force (USPSTF) has revised the eligibility criteria, which may help to ameliorate sex- and race/ethnicity–related disparities in lung cancer screening.

**Objective:**

To determine the changes associated with the revised USPSTF guideline for lung cancer screening eligibility among female, Black, and Hispanic populations using a large nationwide survey.

**Design, Setting, and Participants:**

This cross-sectional study included respondents to the Centers for Disease Control and Prevention’s Behavioral Risk Factor Surveillance System who were 50 to 80 years of age with a smoking history in 19 states that used the optional lung cancer screening module. The change in eligibility among female, male, Black, Hispanic, and White participants was examined. Eligibility by sex and race/ethnicity was compared with a reference population. Data were collected from January 1, 2017, to December 31, 2018, and analyzed from May 8 to June 11, 2020.

**Exposures:**

Self-reported sex, race/ethnicity, age, and smoking history.

**Main Outcomes and Measures:**

Lung cancer screening eligibility using the revised USPSTF criteria. The previous criteria included current or past smokers (within 15 years) who were 55 to 80 years of age and had a smoking history of more than 30 pack-years. In the revised criteria, age was modified to 50 to 80 years; smoking history, to 20 pack-years.

**Results:**

Among 40 869 respondents aged 50 to 80 years with a smoking history, 21 265 (52.0%) were women, 3430 (8.4%) were Black, and 1226 (30.0%) were Hispanic (mean [SD] age, 65.6 [7.9] years). The revised criteria increased eligibility for the following populations: men (29.4% to 38.3% [8.9% difference]; *P* < .001), women (25.9% to 36.4% [10.5% difference]; *P* < .001), White individuals (31.1% to 40.9% [9.8% difference]; *P* < .001), Black individuals (16.3% to 28.8% [12.5% difference]; *P* < .001), and Hispanic individuals (10.5% to 18.7% [8.2% difference]; *P* < .001). The odds of eligibility were lower for women compared with men (adjusted odds ratio [AOR], 0.88; 95% CI, 0.79-0.99; *P* = .04) and for Black (AOR, 0.43; 95% CI, 0.33-0.56; *P* < .001) and Hispanic populations (AOR, 0.70; 95% CI, 0.62-0.80; *P* < .001) compared with the White population.

**Conclusions and Relevance:**

The revised USPSTF guideline may likely increase lung cancer screening rates for female, Black, and Hispanic populations. However, despite these potential improvements, lung cancer screening inequities may persist without tailored eligibility criteria.

## Key Points

**Question:**

What change will be associated with the revised US Preventive Services Task Force (USPSTF) lung cancer screening guideline for screening eligibility among female, Black, and Hispanic populations?

**Findings:**

In this cross-sectional study of the Behavioral Risk Factor Surveillance System for 2017 and 2018, the proportion eligible for screening among current and former smokers increased by 30.3% for men, 40.5% for women, and 31.9% for White, 76.7% for Black, and 78.1% for Hispanic populations. Compared with men, women had lower odds of eligibility, and compared with White, Black and Hispanic individuals had lower odds of eligibility.

**Meaning:**

These findings suggest that screening disparities may persist among women and racial/ethnic minority populations with the revised guideline.

## Teaser

This cross-sectional study assesses the changes associated with the revised US Preventive Services Task Force guideline with lung cancer screening eligibility among female, Black, and Hispanic populations using a large nationwide survey.

## Introduction

In the United States, lung cancer is the leading cause of cancer-related deaths—killing approximately 135 720 people in 2020.^1^ Despite decreasing incidence of and mortality rates due to lung cancer in the population as a whole, certain minority and vulnerable populations remain at elevated risk. For example, Black individuals who smoke continue to have a higher risk of developing and dying from lung cancer with less smoking exposure compared with White smokers,^2^ and incidence rates for women have not improved to the same extent as for men.^3^ Finally, Hispanics are more likely to have advanced stages of lung cancer when diagnosed and are less likely to undergo surgery.^4^

Lung cancer screening can save lives, but the rate of eligibility varies among populations. Since 2013, the US Preventive Services Task Force (USPSTF) has recommended low-dose computed tomography for people who currently smoke or have quit within 15 years, are 55 to 80 years of age, and have a smoking history of more than 30 pack-years.^5^ These criteria were based in part on the 2004 National Lung Screening Trial, in which 53 454 high-risk patients were randomly assigned to low-dose computed tomography or chest radiography. The low-dose computed tomography group had a 20% relative reduction in lung cancer mortality.^6^ Since this trial, researchers have highlighted that certain populations were underrepresented in the National Lung Screening Trial, which may have led to screening eligibility and cancer outcome disparities among those who have a higher risk of lung cancer at a younger age with less smoking exposure.^7^ To reflect current evidence, the USPSTF has revised the lung cancer screening eligibility criteria by lowering the age to 50 years and reducing the smoking history to 20 pack-years, which may help to ameliorate sex- and race/ethnicity-related disparities.^8^

Increasing eligibility across the general population has potential implications for the effectiveness of lung cancer screening. Lowering the threshold for age and smoking exposure can increase the rate of false-positive results and prioritize older smokers with relatively low life expectancies.^9^ Hence, research is needed to investigate how the revised eligibility criteria affect minority and vulnerable populations. The objective of this study was to determine the potential change associated with the revised USPSTF guideline for lung cancer screening eligibility among female, Black, and Hispanic populations using data from a large nationwide survey.

## Methods

### Study Sample

The data for this study were derived from the Centers for Disease Control and Prevention’s Behavioral Risk Factor Surveillance System (BRFSS) survey. The BRFSS is a nationwide yearly telephone survey that collects health and wellness data to inform research, practices, and policies.^10^ This analysis used data from states that administered the optional lung cancer screening module in 2017 (Florida, Georgia, Kansas, Kentucky, Maine, Maryland, Missouri, Nevada, Oklahoma, Vermont, and Wyoming) and 2018 (Delaware, Maine, Maryland, New Jersey, Oklahoma, South Dakota, Texas, and West Virginia).^10^ This secondary analysis was deemed exempt from guidelines for research involving human participants by the University of Utah institutional review board. We followed the Strengthening the Reporting of Observational Studies in Epidemiology (STROBE) reporting guideline.

### Measures

The BRFSS lung cancer screening module was administered to respondents who had smoked at least 100 cigarettes in their lifetime (ever smokers) from January 1, 2017, to December 31, 2018. The lung cancer screening module consisted of 3 questions to determine screening eligibility: the age when regular smoking started, the age when regular smoking ended, and the mean number of cigarettes smoked each day. We calculated the pack-year history by multiplying the mean packs smoked per day by the number of years the respondent smoked. The mean packs smoked per day was calculated by dividing the mean number of cigarettes smoked per day by 20, and the number of years was calculated by subtracting the age when the respondent last smoked from the age that they started. If the current age of the respondent was greater than 15 years from when they last smoked, they were classified as ineligible. Demographic variables included sex, race/ethnicity, income, marital status, educational level, age, and general health. Populations of interest included women, men, Whites, Blacks, and Hispanics. Respondent demographics and smoking exposure were self-reported.

### Statistical Analysis

Data were analyzed from May 8 to June 11, 2020. The USPSTF eligibility criteria are under review in 2020 to include people who are younger (50 vs 55 years) and smoke less (history of 20 vs 30 pack-years).^5^ As such, for each population of interest, we examined the change in eligibility comparing the revised with the original criteria, and we compared the likelihood of eligibility for specific groups with that of a reference population. Weighted percentages and χ^2^ tests of independence with Rao and Scott adjustment^11^ were used to test for differences in eligibility with the revised criteria. We used bivariable and multivariable logistic regression to compare women with men and Black and Hispanic individuals with White individuals. The adjusted odds ratio (AOR) comparing men with women controlled for income, race/ethnicity, marital status, educational level, age, year of survey, and general health. The AOR comparing race/ethnicity controlled for income, sex, marital status, educational level, age, year of survey, and general health. Hypothesis tests were 2 sided, and *P* < .05 indicated statistical significance. We used R, version 3.5.3 (R Foundation for Statistical Computing), with the Survey package to account for the BRFSS complex survey design. The variance was estimated using Taylor series linearization, and outcomes were reported without correcting for multiple comparisons.^12^

## Results

In 2017 and 2018, 19 states used the BRFSS lung cancer screening module (including 3 that used the module both years). A total of 40 869 respondents (19 604 men [48.0%] and 21 265 women [52.0%]) were 50 to 80 years of age (mean [SD] age, 64.6 [7.9] years) and had a history of smoking. Most respondents were White (33 304 [81.5%]), whereas 3430 (8.4%) were Black and 1226 (30.0%) were Hispanic (Table 1).

**Table 1.** Unweighted Characteristics of Adults Who Ever Smoked From 2017-2018 BRFSS

Characteristic	Age group, No. (%)^a^	
	50-54 y (n = 5124)	55-80 y (n = 35 745)
Sex		
Male	2361 (46.1)	17 209 (48.1)
Female	2756 (53.8)	18 509 (51.8)
Smoking status		
Current (everyday)	1769 (34.5)	7241 (20.3)
Current (some days)	600 (11.7)	2619 (7.3)
Former	2744 (53.6)	25 812 (72.2)
Insurance coverage		
Yes	4423 (86.3)	33 840 (94.7)
No	689 (13.4)	1814 (5.1)
Income, $		
<15 000	655 (12.8)	3563 (10.0)
15 000-24 999	722 (14.1)	5480 (15.3)
25 000-34 999	409 (8.0)	3485 (9.7)
35 000-49 999	512 (10.0)	4599 (12.9)
>50 000	2099 (41.0)	12 563 (35.1)
Race/ethnicity		
White, non-Hispanic	4007 (78.2)	29 297 (82.0)
Black, non-Hispanic	437 (8.5)	2993 (8.4)
Hispanic	257 (5.0)	969 (2.7)
Other and multiracial, non-Hispanic	330 (6.4)	1791 (5.0)
Educational status		
Less than high school education	578 (11.3)	3143 (8.8)
Completed high school education	1756 (34.3)	11 318 (31.7)
Some college	1517 (29.6)	10 552 (29.5)
Completed college education	1259 (24.6)	10 646 (29.8)
Marital status		
Married	2640 (51.5)	18 304 (51.2)
Divorced	1192 (23.3)	7639 (21.4)
Widowed	248 (4.8)	5969 (16.7)
Separated	222 (4.3)	785 (2.2)
Never married	622 (12.1)	2235 (6.3)
Member of an unmarried couple	164 (3.2)	625 (1.7)
General health		
Excellent	556 (10.9)	3756 (10.5)
Very good	1427 (27.8)	10 050 (28.1)
Good	1603 (31.3)	11 495 (32.2)
Fair	974 (19.0)	6778 (19.0)
Poor	547 (10.7)	3563 (10.0)

Abbreviation: BRFSS, Behavioral Risk Factor Surveillance System.

^a^ Owing to missing data, numbers may not total those in column heads and percentages may not total 100.

Comparing the revised criteria with the original USPSTF criteria, the proportion of men who were eligible significantly increased from 29.4% (95% CI, 27.6%-31.0%) to 38.3% (95% CI, 36.3%-40.0%; difference, 8.9%; relative increase, 30.3%), and the proportion of women who were eligible increased from 25.9% (95% CI, 24.2%-28.0%) to 36.4% (95% CI, 34.6%-38.0%; difference, 10.5%; relative increase, 40.5%). In racial/ethnic populations, the revised criteria increased eligibility for the following populations: White individuals, 31.1% (95% CI, 29.8%-33.0%) to 40.9% (95% CI, 39.5%-42.0%; difference, 9.8%; relative increase, 31.9%); Black individuals, 16.3% (95% CI, 12.2%-21.0%) to 28.8% (95% CI, 24.0%-34.0%; difference, 12.5%; relative increase, 76.7%); and Hispanic individuals, 10.5% (95% CI, 6.2%-17.0%) to 18.7% (95% CI, 12.6%-27.0%; difference, 8.2%; relative increase, 78.1%) (*P* < .001 for all comparisons) (Table 2). The odds of eligibility for women were lower compared with that for men, using the multivariable logistic regression model (AOR, 0.88; 95% CI, 0.79-0.99; *P* = .04); the odds of eligibility for Black individuals (AOR, 0.43; 95% CI, 0.33-0.56; *P* < .001) and Hispanic individuals (AOR, 0.70; 95% CI, 0.62-0.80; *P* < .001) were lower compared with that of White individuals (Table 3).

**Table 2.** Comparison of USPSTF Lung Cancer Screening Criteria Among Adults Who Ever Smoked

Population	Eligibility criteria^a^					
	Aged 55-80 y and smoking history of 30 pack-years			Aged 50-80 y and smoking history of 20 pack-years		
	No. eligible	Weighted No. eligible	Weighted frequency, % (95% CI)	No. eligible	Weighted No. eligible	Weighted frequency, % (95% CI)
Sex						
Male	3608	778 457	29.4 (27.6-31.0)	5164	1 230 914	38.3 (36.3-40.0)
Female	3210	594 408	25.9 (24.2-28.0)	5224	1 017 652	36.4 (34.6-38.0)
Race/ethnicity						
White, non-Hispanic	5981	1 164 303	31.1 (29.8-33.0)	8932	1 842 033	40.9 (39.5-42.0)
Black, non-Hispanic	257	81 529	16.3 (12.2-21.0)	523	177 638	28.8 (24.0-34.0)
Hispanic	102	42 907	10.5 (6.2-17.0)	198	99 292	18.7 (12.6-27.0)

Abbreviation: USPSTF, US Preventive Services Task Force.

^a^ Difference between eligibility criteria for each population group were significant at *P* < .001.

**Table 3.** Women and Racial/Ethnic Minority Comparison Among Adults Who Ever Smoked Using USPSTF Lung Cancer Screening Criteria

Population	Eligibility criteria							
	Aged 55-80 y and smoking history of 30 pack-years				Aged 50-80 y and smoking history of 20 pack-years			
	OR (95% CI)	*P* value	AOR (95% CI)^a^	*P* value	OR (95% CI)	*P* value	AOR (95% CI)^a^	*P* value
Sex								
Male	1 [Reference]	NA	1 [Reference]	NA	1 [Reference]	NA	1 [Reference]	NA
Female	0.84 (0.74-0.96)	.008	0.81 (0.71-0.92)	.002	0.92 (0.82-1.04)	.17	0.88 (0.79-0.99)	.04
Race/ethnicity								
White, non-Hispanic	1 [Reference]	NA	1 [Reference]	NA	1 [Reference]	NA	1 [Reference]	NA
Black, non-Hispanic	0.43 (0.31-0.61)	<.001	0.31 (0.21-0.46)	<.001	0.59 (0.45-0.76)	<.001	0.43 (0.33-0.56)	<.001
Hispanic	0.71 (0.62-0.81)	<.001	0.65 (0.56-0.76)	<.001	0.76 (0.68-0.85)	<.001	0.70 (0.62-0.80)	<.001

Abbreviations: AOR, adjusted odds ratio (OR); NA, not applicable; USPSTF, US Preventive Services Task Force.

^a^ Controlled for income, race/ethnicity, sex, marital status, educational level, age, year of survey, and general health.

## Discussion

This study examined the change in lung cancer screening eligibility among women and racial/ethnic minority populations with the revised USPSTF guideline. The revised USPSTF guideline will likely increase lung cancer screening rates for female, Black, and Hispanic populations. However, despite these potential improvements, eligibility disparities may persist.

Using the former USPSTF criteria, eligibility among BRFSS respondents aligns with results from a large prospective cohort study^13^ in which 17% of Black smokers were eligible compared with 31% of White smokers. However, the present study results suggest that the relative increase in eligibility among BRFSS respondents may be more conservative than what was anticipated from the Agency for Healthcare Research and Quality Technical Report,^14^ in which multiple models, primarily based on clinical trials, were evaluated. The Agency for Healthcare Research and Quality report provides estimates that eligibility among women would increase by 96%; among men, by 81%; among Black individuals, by 105%; among Hispanic individuals, by 100%; and among White individuals, by 78%.^14^

Results from the present investigation suggest that Black and Hispanic smokers are likely to continue to be underrepresented among individuals eligible for lung cancer screening, despite data suggesting that their risk of lung cancer is equivalent to or greater than that of White smokers. Data indicate that Black smokers are at significantly higher risk for lung cancer at all levels of smoking up to 30 cigarettes per day, at which point the difference is no longer significant.^2^ One potential explanation is that Black smokers take in more nicotine and carcinogens per cigarette than do White smokers, and that the nicotine and carcinogen exposure may be even greater for menthol cigarette smokers compared with regular cigarette smokers, with Black smokers being much more likely to use menthol cigarettes compared with White smokers.^15,16^ Hence, the risk of lung cancer may be elevated for Black compared with White smokers at the same level of exposure because of higher nicotine and carcinogen levels. Moreover, nearly 25% of Hispanics who would be classified as light smokers (1-9 cigarettes per day) may underreport the quantity of cigarettes smoked, which could result in some heavy (≥20 cigarettes per day) or moderate (10-19 cigarettes per day) smokers being misclassified.^17^ Our results suggest that lung cancer screening inequity may persist or worsen for Hispanic individuals compared with White individuals. As such, simply raising or lowering the criteria based on age and smoking history are unlikely to have a meaningful effect on reducing inequities across racial/ethnic groups, and lung cancer screening criteria are likely to remain biased against Black and Hispanic smokers unless the criteria are adapted for different racial/ethnic groups.

### Limitations

Limitations of this study should be noted. First, although the BRFSS is the one of the largest nationwide surveys, the data are cross-sectional. Second, states that opted for the lung cancer screening module may not be representative of the nation. Third, the use of odds ratios may have overestimated the true measure of association. Finally, this analysis evaluated eligibility for lung cancer screening, which may not reflect receipt of a low-dose computed tomographic scan after shared decision-making with a clinician.

## Conclusions

These findings suggest that female, Black, and Hispanic populations remain less likely to be eligible for lung cancer screening with the revised USPSTF guideline. Eligibility criteria may need to be tailored for women and racial and ethnic minority populations to reduce inequities.
